# Feasibility of containing shigellosis in Hubei Province, China: a modelling study

**DOI:** 10.1186/s12879-020-05353-4

**Published:** 2020-09-01

**Authors:** Jia Rui, Qi Chen, Qiuping Chen, Qingqing Hu, Mikah Ngwanguong Hannah, Zeyu Zhao, Yao Wang, Xingchun Liu, Zhao Lei, Shanshan Yu, Yi-Chen Chiang, Benhua Zhao, Yanhua Su, Bin Zhao, Tianmu Chen

**Affiliations:** 1 State Key Laboratory of Molecular Vaccinology and Molecular Diagnostics, School of Public Health, Xiamen University, 4221-117 South Xiang’an Road, Xiang’an District, Xiamen City, Fujian Province People’s Republic of China; 2 Hubei Provincial Center for Disease Control and Prevention, Wuhan City, Hubei Province People’s Republic of China; 3 Medical Insurance Office, Xiang’an Hospital of Xiamen University, Xiamen City, Fujian Province People’s Republic of China; 4 Division of Public Health, School of Medicine, University of Utah, 201 Presidents Circle, Salt Lake City, UT 84112 USA; 5 Medical College, Xiamen University, Xiamen City, Fujian Province People’s Republic of China; 6 Laboratory Department, Xiang’an Hospital of Xiamen University, State Key Laboratory of Molecular Vaccinology and Molecular Diagnosis, Xiamen City, Fujian Province People’s Republic of China

**Keywords:** Shigellosis, Transmission control, Feasibility, Mathematical model

## Abstract

**Background:**

The transmission features and the feasibility of containing shigellosis remain unclear among a population-based study in China.

**Methods:**

A population–based Susceptible – Exposed – Infectious / Asymptomatic – Recovered (SEIAR) model was built including decreasing the infectious period (DIP) or isolation of shigellosis cases. We analyzed the distribution of the reported shigellosis cases in Hubei Province, China from January 2005 to December 2017, and divided the time series into several stages according to the heterogeneity of reported incidence during the period. In each stage, an epidemic season was selected for the modelling and assessing the effectiveness of DIP and case isolation.

**Results:**

A total of 130,770 shigellosis cases were reported in Hubei Province. The median of *R*_*eff*_ was 1.13 (range: 0.86–1.21), 1.10 (range: 0.91–1.13), 1.09 (range: 0.92–1.92), and 1.03 (range: 0.94–1.22) in 2005–2006 season, 2010–2011 season, 2013–2014 season, and 2016–2017 season, respectively. The reported incidence decreased significantly (trend *χ*^2^ = 8260.41, *P* <  0.001) among four stages. The incidence of shigellosis decreased sharply when DIP implemented in three scenarios (*γ* = 0.1, 0.1429, 0.3333) and when proportion of case isolation increased.

**Conclusions:**

Year heterogeneity of reported shigellosis incidence exists in Hubei Province. It is feasible to contain the transmission by implementing DIP and case isolation.

## Background

Globally, *Shigella spp*, which causes severe diarrhea and dysentery, is the second leading cause of diarrhea death following rotavirus, and it is estimated that the pathogen causes approximately 210,000 deaths among all ages, including about 63,700 children under the age of five [1]. Despite mortality from diarrhea has declined, its incidence remains high, particularly in economically underdeveloped countries [2, 3]. The high-risk groups include children aged 1 to 4 years old, and some other groups include travelers to areas where dysentery is prevalent and men who have sex with men [2, 4–7]. Because humans are the only host of *Shigella spp*, diarrhea is transmitted by contact between people and some related life behaviors, while food and water are less common to transmit diarrhea. In addition, flies can spread the pathogen in environments where feces pollute the environment [8, 9]. Because asymptomatic individuals are unpredictable, the disease burden on the disease is particularly severe worldwide.

At present, the main prevention and control measures of shigellosis include managing the sources of infection, cutting off the transmission route and protecting the susceptible population [10–12]. Early detection of patients and carriers, timely isolation and thorough treatment are important measures to control shigellosis. The ways of cutting off transmission way includes managing water, excrement and food, managing the transmission through flies and washing hands before eating and after using the toilet [13].

The quantitative prediction and early warning of epidemic situation based on the model has become the focus of the public health field, and more quantitative prediction data have been gradually added to the qualitative assessment of trend judgment. The ARIMA model, GM (1,1) gray model, prospective space-time scan statistic, Markov model and mathematical model such as a waterborne pathogen model termed the Susceptible–Infectious–Recovered–Water (SIRW) model, are commonly used to forecast the incidence of bacillary dysentery [9, 14–16]. Considering the asymptomatic infection, we previously built a Susceptible–Exposed–Infectious/Asymptomatic–Recovered–Water (SEIARW) model to simulate the transmission and to assess the effectiveness of the key interventions in a small-scale outbreak in a school [14]. However, the transmission features and the feasibility of containing the transmission remain unclear among a whole population in a large outbreak in China. According to our previous researches [14, 17], Susceptible – Exposed – Infectious / Asymptomatic – Recovered – Water (SEIARW) model, in which two routes (person–to–person and reservoir–to–person) were considered, could be used to simulate the enteric infectious diseases including shigellosis. However, the latest research showed that shigellosis transmits primarily from person–to–person [2]. Considering the high coverage of the municipal water systems which provide the disinfected water in China and reservoir–to–person transmission only occasionally reported in small scale outbreak in schools in rural areas [18, 19], we developed a whole–population–based Susceptible – Exposed – Infectious / Asymptomatic – Recovered (SEIAR) model (denoted as Model 1) which only includes the transmission route of person–to–person [20–22].

This study collected data on the incidence of bacterial dysentery in Hubei Province. The aim was to find the better prevention and control measures by simulating the effectiveness of symptomatic infection and simulating the effectiveness of different isolation rates, so as to reduce the disease burden.

## Methods

### Study design

We conducted a time series study in shigellosis cases reported in Hubei Province from January 2005 to November 2017. We performed a modelling study to simulate the incidence of the transmission and to assess the effectiveness of intervention to contain the transmission in the area.

This effort of disease control was part of CDC’s routine responsibility in Hubei Province; therefore, institutional review and informed consent were not required for this study. All data analyzed were anonymized.

### Data collection

Hubei Province, locating at the north of the Dongting Lake and in the central of China, has a population of more than 58 million. This study was based on a dataset of reported Shigellosis cases was built from January 2005 to December 2017 in the province. The illness onset date of each case was included in the data. Cases were reported from doctors in clinics or hospitals in Hubei province and were identified following the case definitions with three categories: 1) Suspected cases; 2) Clinically diagnosed cases; 3) Confirmed cases, which were based on the “Diagnostic criteria for bacterial and amoebic dysentery (WS287-2008)” announced by the National Health Commission of the People’s Republic of China. The detailed definitions of the three categories above can be consulted from existing literature [23]. In this study, we included clinically diagnosed cases and confirmed cases for the analysis.

### The transmission models

In the model, people were divided into susceptible (*S*), exposed (*E*), infectious (*I*), asymptomatic (*A*), and recovered (*R*) individuals. The equations of the model are as follows:

$$ \frac{ds}{dt}=- bs\left(i+ ka\right) $$

$$ \frac{de}{dt}= bs\left(i+ ka\right)-\omega e $$

$$ \frac{di}{dt}=\left(1-p\right)\upomega e-\gamma i $$

$$ \frac{da}{dt}=p\upomega e-{\gamma}^{\prime }a $$

$$ \frac{dr}{dt}=\gamma i+{\gamma}^{\prime }a $$

In the model, *N* is assumed to denote the total population size and *s* = *S*/*N*, *e* = *E*/*N*, *i* = *I*/*N*, *a* =*A*/*N*, *r* = *R*/*N*, and *b* = *βN*. The parameters *β*, *k*, *ω*, *p*, *γ*, and *γ’* are transmission relative rate, relative transmissibility of asymptomatic to symptomatic individuals, incubation relative rate, proportion of asymptomatic individuals, infectious period relative rate of symptomatic individuals, and infectious period relative rate of asymptomatic individuals, respectively.

Because of the interventions or the decreasing proportion of susceptible individuals due to the spread of the pathogen and other reasons providing the difficulty to estimate basic reproduction number (*R*_0_), which is defined as the expected number of secondary infections that result from introducing a single infected individual into an otherwise susceptible population [17, 24–26], effective reproduction number (*R*_*eff*_) is commonly employed instead [27]. From the definition, it is clear that when *R*_*eff*_ > 1, the disease is able to spread in the population. If *R*_*eff*_ < 1, the infection will be cleared from the population.

In the Model 1, *R*_*eff*_ was calculated by the equation as follows:

$$ {R}_{eff}= bs\left(\frac{1-p}{\gamma }+\frac{kp}{\gamma^{\prime }}\right) $$

### Decreasing the infectious period

Asymptomatic individuals were not able to be monitored commonly because of lacking relative symptoms including diarrhea, fever, etc. In this study, we simulated the effectiveness of decreasing the infectious period (DIP) of symptomatic individuals. DIP depends on the following conditions: 1) infected individuals would go to hospitals or clinics as soon as possible when they get the symptoms of the infection; 2) the ability of the hospitals or clinics to diagnose and treat the infection (giving the sensitive antibiotics to control the infection). Obviously, the earlier the infected individuals diagnosed and treated, the shorter the infectious period (IP) would be. We simulated the mixed effectiveness of DIP in three scenarios: 1) IP = 10 days (*γ* = 0.1); 2) IP = 7 days (*γ* = 0.1429); and 3) IP = 3 days (*γ* = 0.3333) using Model 1.

### Case isolation

**Fig. 1:**
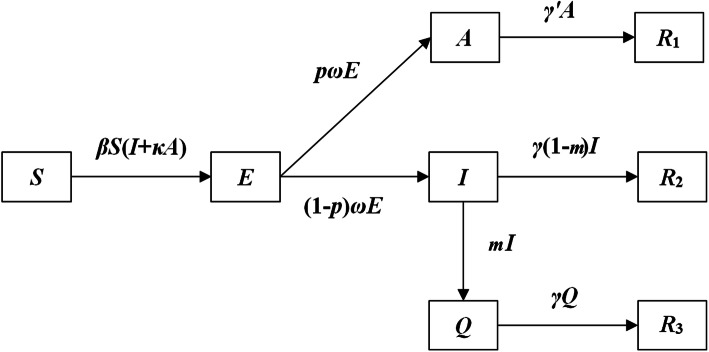
The flowchart of SEIARQ model

In this study, we simulated the effectiveness of case isolation. When cases were diagnosed, the intervention was implemented by the following: 1) the severe cases were isolated in hospital; and 2) the mild cases were quarantined immediately at home and a primary public health provider would perform follow-up visits and provide guidance on quarantine, concurrent disinfection, and terminal disinfection. Because asymptomatic individuals could not been monitored, we assumed that case isolation was only focused on symptomatic individuals. Therefore, we built a Susceptible – Exposed – Infectious/Asymptomatic – Recovered – Quarantined (SEIARQ) model in which quarantined individuals was denoted as *Q*. We set *q* = *Q*/*N*, and *r*_1_, *r*_2_, and *r*_3_ refer to recovered individuals moved from *A*, *I*, and *Q* populations, respectively. The flowchart of SEIARQ model (Model 2) was shown in Fig. 1 and the equations of the model are as follows:

$$ \frac{ds}{dt}=- bs\left(i+ ka\right) $$

$$ \frac{de}{dt}= bs\left(i+ ka\right)-\omega e $$

$$ \frac{di}{dt}=\left(1-p\right)\upomega e-\left(1-m\right)\gamma i- mi $$

$$ \frac{da}{dt}=p\upomega e-{\gamma}^{\prime }a $$

$$ \frac{dq}{dt}= mi-\gamma q $$

$$ \frac{d{r}_1}{dt}={\gamma}^{\prime }a $$

$$ \frac{d{r}_2}{dt}=\left(1-m\right)\gamma i $$

$$ \frac{d{r}_3}{dt}=\gamma q $$

Although *m* represents the isolation coefficient in the model, it is not an isolation ratio. In this study, we define *x* as the isolation ratio calculation based on the final actual isolation cases (*r*_3_) and non-isolated cases (*r*_2_). Since isolation was only focused on cases who had symptoms (*i*) excluding asymptomatic, *r*_1_ was excluded from the calculation of *x*. The calculation formula of *x* was shown as follows:

$$ x=\frac{r_3}{r_2+{r}_3} $$

We simulated 10 scenarios (*x* = 0.1, 0.2, …, 1.0) in which *x* referred to the proportion of casa isolation.

### Indicator developed to assess the effectiveness of interventions

We developed percentage of reduction (PR) under different intervention scenarios to assess the effectiveness of DIP and case isolation. The equation to calculate PR was shown as follows:

$$ {PR}_i=\frac{I_0-{I}_i}{I_0}\times 100\% $$

In the equation, *PR*_*i*_, *I*_0_, and *I*_*i*_ refer to percentage of reduction under different intervention scenarios, incidence of shigellosis under the condition that no intervention was adopted, incidence of shigellosis under the condition that four intervention scenarios were simulated, respectively.

Considering there is no standard threshold of PR to judge the satisfying of the intervention, we simulated PR at 50, 60, 70, 80, and 90% levels.

### Parameter estimation

There are eight parameters (*b*, *k*, *ω*, *p*, *γ*, *γ’* and *m*) in the above models. According to our previous research [14], *k*, *ω*, *p*, *γ*, and *γ’* are disease-specific parameters which could be estimated from literatures. The incubation period of Shigellosis is 1–4 days [2, 28], and commonly 1 days, therefore, *ω* = 1.0. The proportion of asymptomatic infection ranges from 0.0037 to 0.27 [29–31], and can be set *p* = 0.1. The infectious period of symptomatic infection is 13.5 days [14], therefore, *γ* = 0.0741. According to our previous research [14], the infectious period of asymptomatic infection could be simulated 5 weeks in our model, thus *γ’* = 0.0286. Due to reduction of shedding frequency, the relative transmissibility of asymptomatic individual (*k*) was modeled to be a reduced quantity (0.3125) [14]. We set different values of *m* until we got the ten target values of *x*. However, *b* is scenario- or area-specific parameter which is various in different outbreaks even in different periods. Therefore, the parameter is confirmed by curve fitting by Model 1 to the collected data.

### Simulation method and statistical analysis

In this study, we firstly analyzed the temporal distribution of the reported shigellosis cases, and divided the time series into several stages according to the homogeneity of reported incidence during the period. In each stage, an epidemic season was selected for the modelling and assessing the effectiveness of the interventions of DIP and case isolation.

Berkeley Madonna 8.3.18 (developed by Robert Macey and George Oster of the University of California at Berkeley. Copyright©1993–2001 Robert I. Macey & George F. Oster) was employed for model simulation. Least root mean square (LRMS) and determination coefficient (*R*^2^) were adopted to judge the goodness of fit. The simulation methods were the same as the previously published researches [14, 17, 24, 32, 33]. The chi-square test was performed by SPSS 13.0 (IBM Corp., Armonk, NY, USA).

## Results

### Basic characteristics of the reported cases

**Fig. 2:**
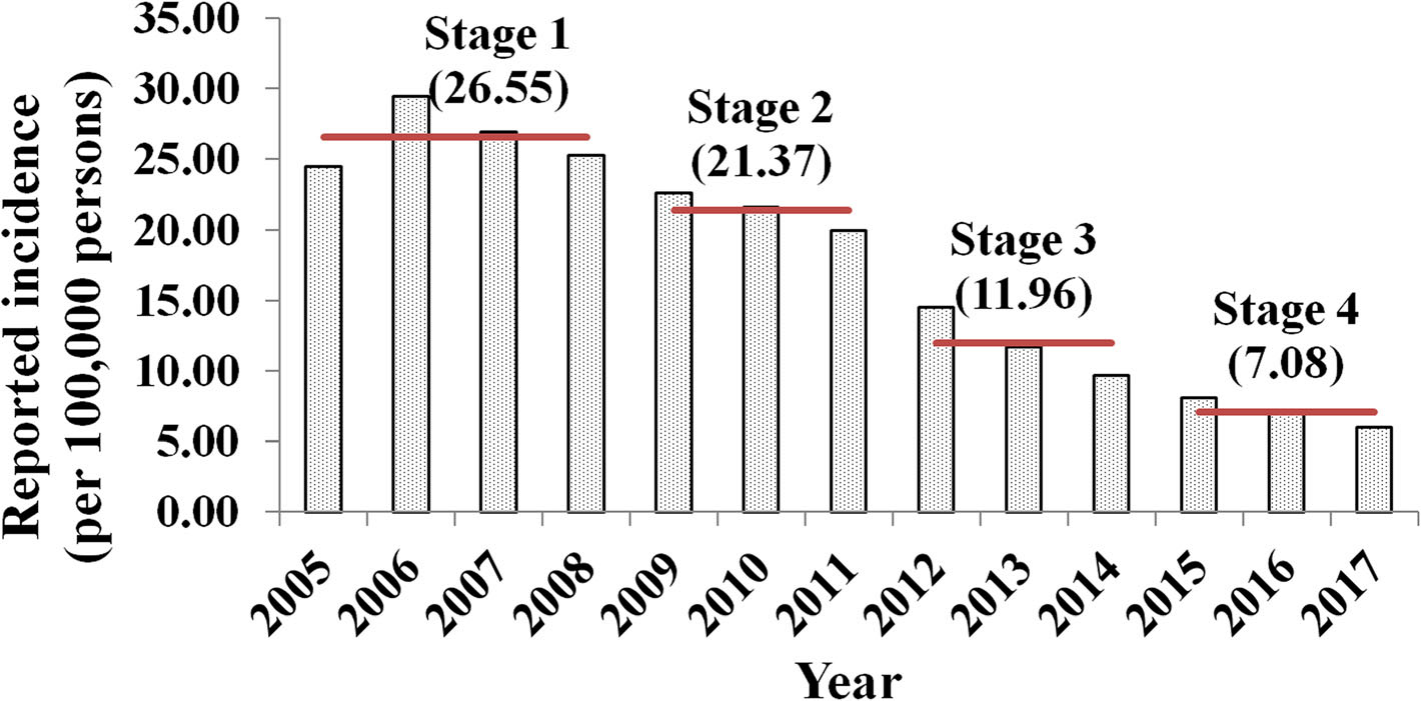
Reported incidences and stages of shigellosis transmission from 2005 to 2017

During the study period, 130,770 shigellosis cases were reported in Hubei Province. According to the yearly incidence of the disease, the study period was divided into four stages: 1) stage 1 was from 2005 to 2008; 2) stage 2 was from 2009 to 2011; 3) stage 3 was from 2012 to 2014; and 4) stage 4 was from 2015 to 2017. The reported incidence decreased significantly (trend *χ*^2^ = 8260.41, *P* <  0.001) among the four stages (Fig. 2).

### Model fitting

**Fig. 3:**
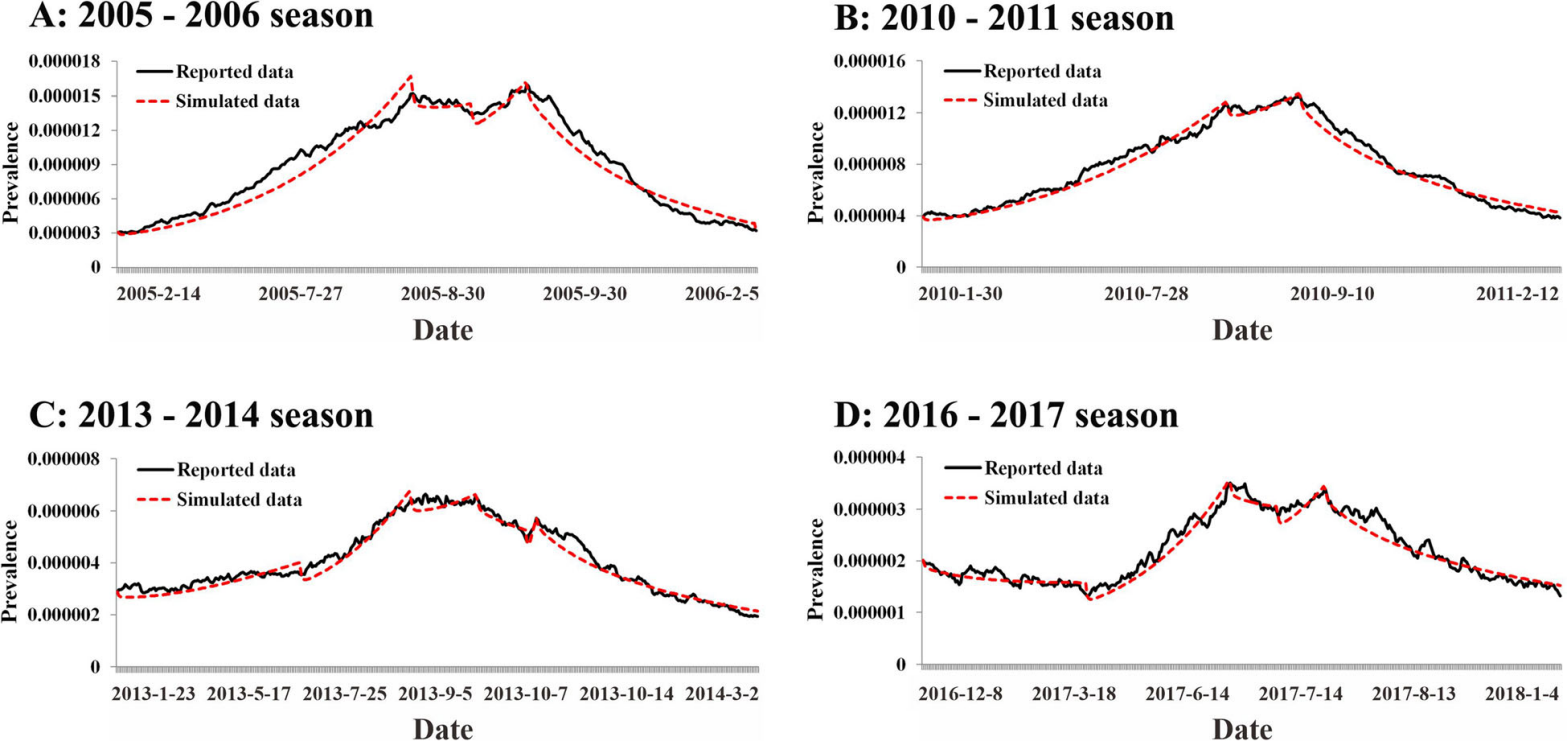
The results of model fitting of SEIAR model to reported data in different stages. **a**, Curve fitting in 2005–2006 season; **b**, Curve fitting in 2010–2011 season; **c**, Curve fitting in 2013–2014 season; **d**, Curve fitting in 2016–2017 season

One epidemic season, which is the time span between two lowest values of daily reported incidence during a year, was selected from each stage for the simulation (Table 1). By model fitting and the rule of LRMS, each selected epidemic season was divided into several sub-seasons (Fig. 3). The model fitted the data well except sub-season 2 in 2005–2006 season and sub-season 3 in 2013–2014 season (Table 1). The median of *R*_*eff*_ was 1.13 (range: 0.86–1.21), 1.10 (range: 0.91–1.13), 1.09 (range: 0.92–1.92), and 1.03 (range: 0.94–1.22) in 2005–2006 season, 2010–2011 season, 2013–2014 season, and 2016–2017 season, respectively.

**Table 1:** Basic features of the selected data for the simulation

Stages	Epidemic seasons selected	Sub-seasons included			Number of reported cases included	*b*	*R*_*eff*_	*R*^2^	*P*
		ID^a^	Start time	Stop time					
Stage 1	2005–2006	1	February 14, 2005	July 26, 2005	6189	0.0900	1.19	0.94	< 0.001
		2	July 27, 2005	August 28, 2005	1917	0.0805	1.07	0.01	0.5570
		3	August 29, 2005	September 27, 2005	1917	0.0911	1.21	0.87	< 0.001
		4	September 28, 2005	February 2, 2006	3684	0.0649	0.86	0.98	< 0.001
Stage 2	2010–2011	1	January 31, 2010	July 28, 2010	5896	0.0853	1.13	0.97	< 0.001
		2	July 29, 2010	September 9, 2010	2321	0.0833	1.10	0.73	< 0.001
		3	September 10, 2010	February 11, 2011	4305	0.0689	0.91	0.99	< 0.001
Stage 3	2013–2014	1	January 23, 2013	May 16, 2013	1643	0.0818	1.08	0.78	< 0.001
		2	May 17, 2013	July 23, 2013	1558	0.0912	1.21	0.97	< 0.001
		3	July 24, 2013	September 2, 2013	1132	0.0826	1.09	0.07	0.0917
		4	September 3, 2013	October 3, 2013	675	0.0748	0.99	0.89	< 0.001
		5	October 4, 2013	October 9, 2013	164	0.1449	1.92	0.75	0.0266
		6	October 10, 2013	February 25, 2014	1826	0.0694	0.92	0.98	< 0.001
Stage 4	2016–2017	1	December 8, 2016	March 17, 2017	694	0.0758	1.00	0.54	< 0.001
		2	March 18, 2017	June 12, 2017	953	0.0925	1.22	0.95	< 0.001
		3	June 13, 2017	July 11, 2017	381	0.0774	1.03	0.69	< 0.001
		4	July 12, 2017	August 9, 2017	412	0.0910	1.20	0.78	< 0.001
		5	August 10, 2017	December 31, 2017	1247	0.0712	0.94	0.94	< 0.001

^*a*^*ID* Identification, *b* Transmission relative rate, *R*_*eff*_ Effective regeneration coefficient

### Effectiveness of DIP

**Fig. 4:**
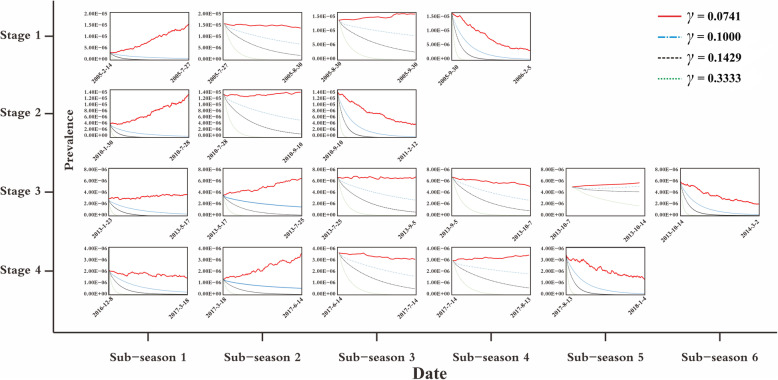
The simulated incidence of different DIP scenarios in different stages. (*γ, infectious period relative rate of symptomatic individuals)

The incidence of shigellosis decreased sharply with the decrease of the infectious period through simulating the effectiveness of DIP in three scenarios (*γ* = 0.1, 0.1429, 0.3333) among 18 sub-seasons (Fig. 4).

### Effectiveness of case isolation

**Fig. 5:**
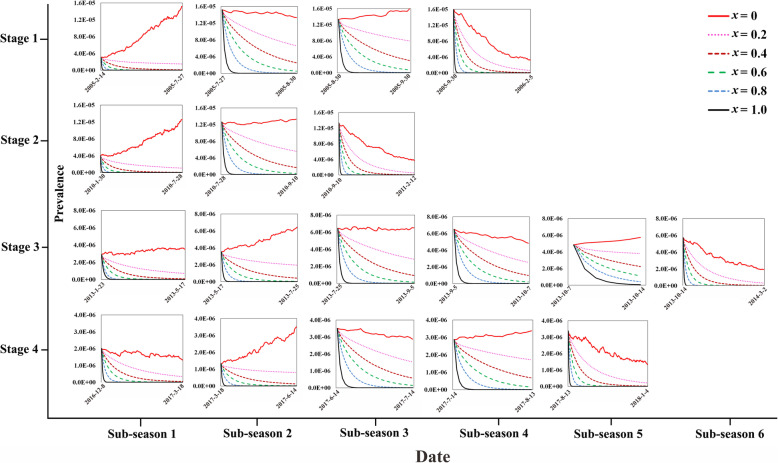
The simulated incidence of different case isolation scenarios in different stages. (*x, actual isolation ratio)

**Fig. 6:**
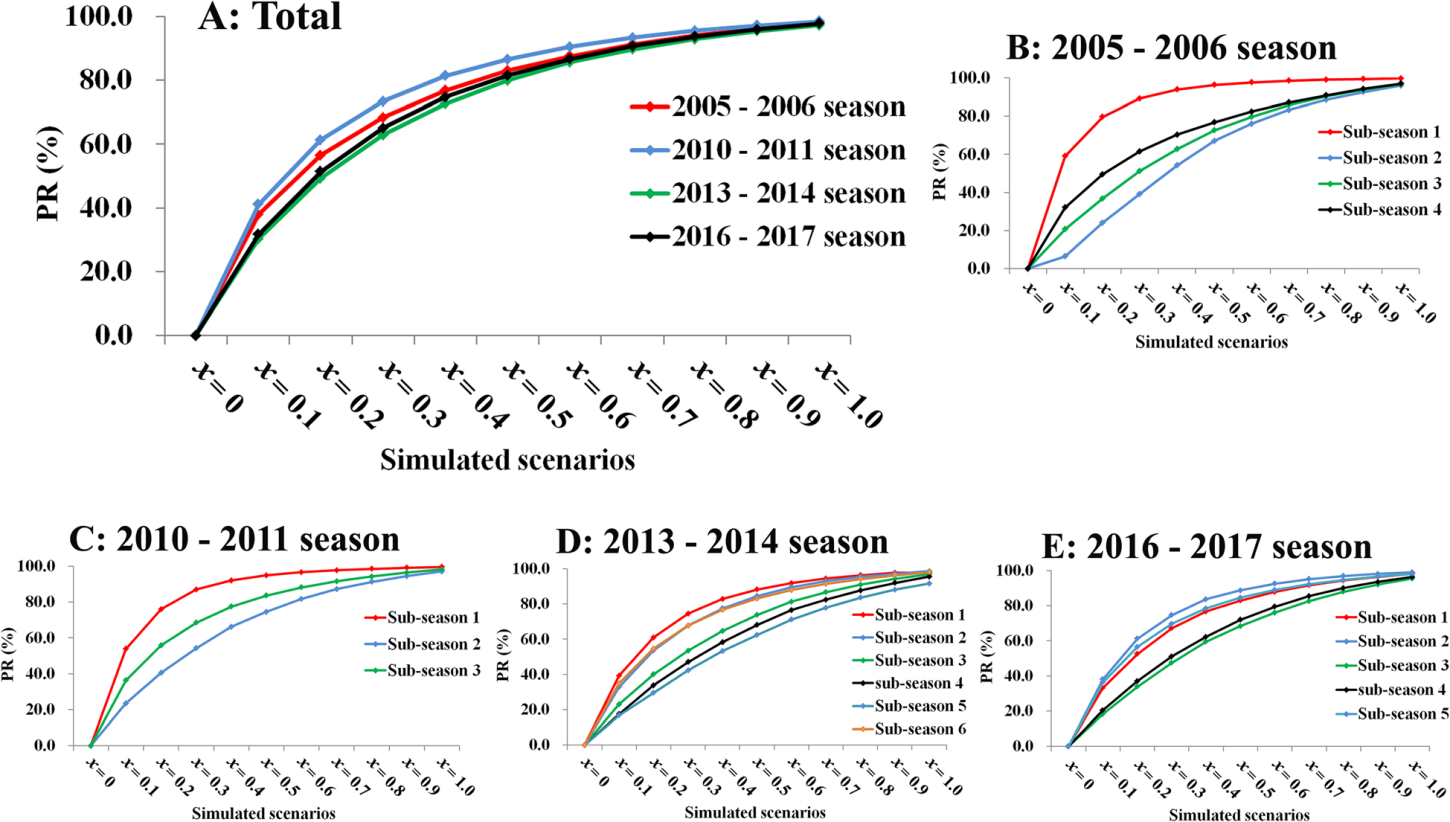
The simulated PR values of different case isolation scenarios in different epidemic seasons. **a**, total; **b**, 2005–2006 season; **c**, 2010–2011 season; **d**, 2013–2014 season; **e**, 2016–2017 season. (*PR, percentage of reduction)

By simulating 10 case isolation scenarios (*x* = 0.1, 0.2, …, 1.0) among 18 sub-seasons, the incidence of shigellosis decreased much sharply when *x* increased (Fig. 5). Higher value of *x* provided higher PR in each sub-season (Fig. 6). The value of *x* was 0.25 (range: 0.1–0.4), 0.3 (range: 0.2–0.5), 0.4 (range: 0.2–0.6), 0.55 (range: 0.3–0.8), and 0.75 (range: 0.4–1.0), respectively to reach the PR levels of 50–90% (Table 2).

**Table 2:** The values of *x* under different PR levels

PR(%)	Sub-seasons in 2005–2006				Sub-seasons in 2010–2011			Sub-seasons in 2013–2014						Sub-seasons in 2016–2017					Median (range)
	1	2	3	4	1	2	3	1	2	3	4	5	6	1	2	3	4	5	
50	0.1	0.4	0.3	0.3	0.1	0.3	0.2	0.2	0.2	0.3	0.4	0.4	0.2	0.2	0.2	0.4	0.3	0.2	0.25 (0.1–0.4)
60	0.2	0.5	0.4	0.3	0.2	0.4	0.3	0.2	0.3	0.4	0.5	0.5	0.3	0.3	0.2	0.5	0.4	0.3	0.3 (0.2–0.5)
70	0.2	0.6	0.5	0.4	0.2	0.5	0.4	0.3	0.4	0.5	0.6	0.6	0.4	0.4	0.3	0.6	0.5	0.4	0.4 (0.2–0.6)
80	0.3	0.7	0.7	0.6	0.3	0.6	0.5	0.4	0.5	0.6	0.7	0.8	0.4	0.5	0.4	0.7	0.7	0.5	0.55 (0.3–0.8)
90	0.4	0.9	0.8	0.8	0.4	0.8	0.7	0.6	0.7	0.8	0.9	1	0.7	0.7	0.6	0.9	0.8	0.7	0.75 (0.4–1.0)

*PR Percentage of reduction

## Discussion

In recent years, more and more prediction methods and models have been applied to the early warning analysis of infectious diseases [2, 3]. Therefore, the use of various methods to explore the occurrence and development of infectious diseases has been widely valued. This study, based on the incidence of shigellosis in Hubei Province from January 2005 to December 2017, we used the SEIAR model to simulate the effectiveness of reducing the infection period (DIP) of symptomatic individuals, and built the SEIARQ model to simulate the effectiveness of case isolation to find the best prevention and control measures. All of our models have been tested for goodness of fit, the results showed that more than 90% of *R*^2^ are statistically significant, indicating the models have good applicability.

The results showed that the incidence of shigellosis decreased from 2005 to 2017, and could be divided into four stages. The decreased trend revealed that the incidence of the disease might decrease in the following years. Totally, 22 sub-seasons of four epidemic seasons (2005–2006 season, 2010–2011 season, 2013–2014 season, and 2016–2017 season) were selected for the simulating and assessing the effectiveness of DIP and case isolation. The results showed that the prevalence of the disease decreased sharply with DIP form 3 days (*γ* = 0.3333) to 13.5 days (*γ* = 0.0741) in the 22 sub-seasons. The results of the modelling also showed that the prevalence of the disease decreased sharply with the proportion of case isolation from 0% (*x* = 0.1) to 100% (*x* = 1) in the 22 sub-seasons. If we aimed to reach the PR levels of 50, 25% (range: 10–40%) of cases should be isolated. If we aimed to reach the PR levels of 90, 75% (range: 40–100%) of cases should be isolated. Therefore, case isolation and DIP interventions has high feasibility and effectiveness, and we strongly recommended to control the transmission of shigellosis.

The actual isolation ratio *x* is affected by several aspects [34, 35]: 1) the sensitivity of the surveillance system which could monitor the cases in time when the symptoms onset; 2) After diagnosed, according to the severity of the disease, mild patients were generally recommended to be isolated at home, resulting in fewer patients undergoing effective isolation in the hospital.

In our previous research, an outbreak investigation was conducted in a school [14], but it was not investigated in the whole population, this study can provide relevant recommendations for the prevention and control of shigellosis in the whole population. Compared with some previous studies, although there are many epidemiological reports, but there are few reports on the ability to quantify the spread of shigellosis. Our research quantitatively evaluates the spread of shigellosis through mathematical modeling, and the effectiveness of interventions, thus providing a basis for relevant departments to make more appropriate prevention and control decisions.

### Limitations

Our modeling on simulating countermeasures was based on the whole population. However, we did not consider the age-, sex- or area-specific situations. Another limitation is that we did not divide the interval between symptom onset and notification from IP.

## Conclusions

Year heterogeneity of reported shigellosis incidence exists in Hubei Province, China. DIP and case isolation interventions have high effectiveness to control the transmission of shigellosis.

## Data Availability

The datasets used and analyzed during the current study are available from Dr. Qi Chen (chenqi8700@qq.com) on reasonable request.

## Abbreviations

SEIAR: Susceptible – Exposed – Infectious / Asymptomatic – Recovered
DIP: Decreasing the infectious period
SIRW: Susceptible–Infectious–Recovered–Water
SEIARW: Susceptible–Exposed–Infectious/Asymptomatic–Recovered–Water
CISDCP: China Information System for Disease Control and Prevention
*R*_0_: Basic reproduction number
*R*_*eff*_: Effective reproduction number
IP: Infectious period
SEIARQ: Susceptible – Exposed – Infectious / Asymptomatic – Recovered – Quarantined
PR: Percentage of reduction
LRMS: Least root mean square
*R*^2^: Determination coefficient
